# PpSP32, the *Phlebotomus papatasi* immunodominant salivary protein, exerts immunomodulatory effects on human monocytes, macrophages, and lymphocytes

**DOI:** 10.1186/s13071-022-05627-7

**Published:** 2023-01-02

**Authors:** Cyrine Souissi, Soumaya Marzouki, Ines Elbini-Dhouib, Jed Jebali, Fabiano Oliveira, Jesus G. Valenzuela, Najet Srairi-Abid, Shaden Kamhawi, Melika Ben Ahmed

**Affiliations:** 1 Laboratory of Transmission, Control and Immunobiology of Infections (LTCII), LR11IPT02, Pasteur Institute de Tunis, Tunis, Tunisia; 2 Laboratory of Biomolecules, Venoms and Theranostic Applications, LR20IPT01, Pasteur Institute of Tunis, University of Tunis El Manar, 1002 Tunis, Tunisia; 3 Vector Molecular Biology Section, Laboratory of Malaria and Vector Research, National Institute of Allergy and Infectious Diseases, National Institute of Health, Rockville, MD USA; 4 Faculty of Medicine de Tunis, University of Tunis El Manar, Tunis, Tunisia

**Keywords:** *Phlebotomus papatasi*, Leishmaniasis, Saliva, PpSP32, Immunomodulatory effects

## Abstract

**Background:**

The saliva of sand flies, vectors of *Leishmania* parasites, contains several components that exert pharmacological activity facilitating the acquisition of blood by the insect and contributing to the establishment of infection. Previously, we demonstrated that PpSP32 is the immunodominant salivary antigen in humans exposed to *Phlebotomus papatasi* bites and validated its usefulness as a predictive biomarker of disease. PpSP32, whose functions are little known to date, is an intriguing protein due to its involvement in the etiopathogenesis of pemphigus, an auto-immune disease. Herein, we aimed to better decipher its role through the screening of several immunomodulatory activity either on lymphocytes or on monocytes/macrophages.

**Methods:**

Peripheral mononuclear cells from healthy volunteers were stimulated with anti-CD3/anti-CD28 antibodies, phytohemagglutinin, phorbol 12-myristate 13-acetate/ionomycin, or lipopolysaccharide in the presence of increasing doses of PpSP32. Cell proliferation was measured after the addition of tritiated thymidine. Monocyte activation was tested by analyzing the expression of CD86 and HLA-DR molecules by flow cytometry. Cytokine production was analyzed in culture supernatants by ELISA. THP-1-derived macrophages were stimulated with LPS in the presence of increasing doses of PpSP32, and cytokine production was analyzed in culture supernatants by ELISA and multiplex technique. The effect of PpSP32 on NF-kB signaling was tested by Western blot. The anti-inflammatory activity of PpSP32 was assessed in vivo in an experimental inflammatory model of carrageenan-induced paw edema in rats.

**Results:**

Our data showed that PpSP32 down-modulated the expression of activation markers in LPS-stimulated monocytes and THP1-derived macrophages. This protein negatively modulated the secretion of Th1 and Th2 cytokines by human lymphocytes as well as pro-inflammatory cytokines by monocytes, and THP1-derived macrophages. PpSP32 treatment led to a dose-dependent reduction of IκB phosphorylation. When PpSP32 was injected into the paw of carrageenan-injected rats, edema was significantly reduced.

**Conclusions:**

Our data indicates that PpSP32 induces a potent immunomodulatory effect on monocytes and THP-1-derived macrophages. This inhibition could be mediated, among others, by the modulation of the NF-kB signaling pathway. The anti-inflammatory activity of PpSP32 was confirmed in vivo in the carrageenan-induced paw edema model in rats.

**Graphical Abstract:**

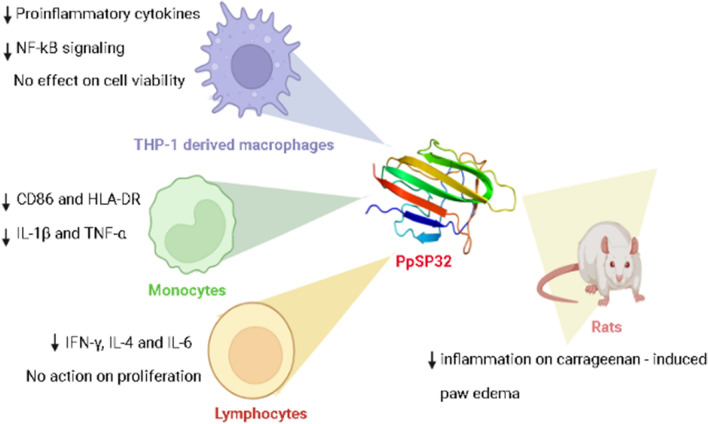

## Background

The constraints imposed on hematophagous insects by the hemostatic and inflammatory response of vertebrates have led insects to put in place during their evolution several strategies to evade the host response. These are mainly through the production of a diversity of salivary proteins with various functions aiming to improve the blood meal while helping to thwart the inflammatory responses of the host [1, 2]. Consistently, the saliva of sand flies, the vectors of leishmaniasis, contains a repertoire of components that exert different pharmacological activity facilitating the acquisition of the blood meal, such as potent anti-hemostatic and vasodilator compounds and immunomodulatory molecules that contribute to the establishment of the infection [2–7]. For instance, maxadilan and adenosine found in the saliva of *Lutzomyia longipalpis* and *Phlebotomus papatasi*, respectively, act as potent vasodilators [8, 9]. A number of immunomodulatory molecules have also been reported in the saliva of sand flies. Such molecules can act on different effector cells and mediators of the immune system [2, 10]. They can alter the function of antigen-presenting cells such as macrophages and dendritic cells [11, 12] and/or affect the expression of co-stimulatory molecules on their surface [13]. They can also alter the complement system by inhibiting both the classical and alternative complement pathways [14]. Furthermore, they can modulate adaptive immune response by disturbing the Th1/Th2 balance. Accordingly, the whole salivary gland lysate of *Lu. longipalpis* downregulates the production of Th1 cytokines and upregulates the production of Th2 cytokines by human peripheral blood mononuclear cells (PBMC) [15]. Hence, the study of the structure and functions of salivary proteins of different vectors has aroused the interest of several researchers over the past two decades [7, 10, 16, 17].

*Phlebotomus papatasi* is the vector of zoonotic cutaneous leishmaniasis, a parasitic disease caused by *Leishmania major* widespread in the Old World mainly in North Africa and Middle East [18]. The salivary gland transcriptome of a colonized Tunisian strain of *P. papatasi* showed the presence of 10 families of salivary proteins, namely the family of odorant-binding proteins (OBP/D7), the family of yellow proteins, the family of antigen 5 proteins, the apyrase, the salivary proteins 32-like (SP32-like) or silk-related proteins, the family of SP16 proteins, 2.5 kDa proteins, the family of 38.8/Aegyptin-like proteins, and the family of Lufaxin-like or SP34 [19]. The family of Sp32-like proteins, initially characterized in the salivary glands of *P. papatasi*, is similar to the silk protein of *Nephila clavipes* [20]. This protein family is specific to sand flies [21]. A member of this family, the salivary protein PpSP32 of *P. papatasi*, has been shown to be the immunodominant target of the antibody response in individuals living in areas endemic for cutaneous leishmaniasis [22]. PpSP32 has homology with collagen adhesion proteins and is predicted to be a mucin based on the pattern of its O- and N-glycosylation [21]. Considering such features, we hypothesized and demonstrated that PpSP32 binds to specific proteins from the skin, desmogleins, leading to the loss of tolerance and the production of related autoimmune antibodies. The latter event may be the first step of the development of an autoimmune disease, pemphigus [23]. PpSP32 is hence an intriguing protein whose functions remain unknown to date. Herein, we aimed to better decipher its functions through the screening of several immunomodulatory activity either on lymphocytes or on monocytes/macrophages.

## Methods

### Samples and cell lines

Peripheral blood samples were collected from 15 healthy volunteers (10 female and 5 male, age range 20–38 years, median, 28 years), with no history of leishmaniasis or travel to sand fly-endemic areas.

THP-1 cells, a human monocytic leukemia cell line, were obtained from the American Type Culture Collection (ATCC) and were maintained in RPMI 1640/Glutamax-1 medium (Invitrogen Life Technologies) supplemented with 10% heat-inactivated fetal bovine serum (Gibco), 1% sodium pyruvate (Gibco), 2% nonessential amino acids (Sigma), 100 U/ml penicillin, 100 µg/ml streptomycin (Gibco), and incubated at 37 °C in a humidified atmosphere consisting of 5% CO_2_.

To induce monocyte/macrophage differentiation, THP-1 cells were seeded in six-well tissue culture plates at 5 × 10^5^ viable cells per well and treated with 20 ng/ml of phorbol 12-myristate 13-acetate (PMA) (Sigma) for 72 h at 37 °C, 5% CO_2_. Differentiated cells were then washed three times with RPMI 1640 and placed in complete media for 24 h at 37 °C, 5% CO_2_.

### Culture media and reagents

PBMCs were cultured in RPMI 1640 medium (Capricorn) supplemented with 10% AB human serum (Sigma), 1% sodium pyruvate 100 mM (Gibco), 1% nonessential amino acids (Gibco), 1% HEPES buffer (Gibco), 0.5% of β-mercaptoethanol 10^−2^ M (Gibco), and 0.2% of 10 mg/ml gentamicin (Gibco).

The recombinant form of PpSP32 was produced as described previously [22]. The following monoclonal antibodies were used for flow cytometry analysis: fluorescein isothiocyanate (FITC), allophycocyanin (APC), and phycoerythrin (PE) conjugated with anti-cluster of differentiation (CD)14, anti-CD86, and human leukocyte antigen receptor (HLA-DR) antibodies, respectively, (BD Biosciences).

### Cell proliferation assay

The PBMCs were isolated on a Ficoll-Hypaque gradient, then cultured in 96-well plates (10^5^ cells/well) for 72 h in a 5% CO_2_ humidified atmosphere at 37 °C with anti-CD3 at 2 μg/ml and anti-CD28 at 2 μg/ml, phytohemagglutinin (PHA) at 10 μg/ml or PMA/ionomycin at 50 ng/ml and 1 μg/ml, respectively, in the presence or absence of different concentrations of the recombinant protein PpSP32 (0.5 μg/ml, 2 μg/ml, 5 μg/ml). All experiments were performed in triplicate. For proliferation studies, the uptake of (3H) thymidine (Amersham) was measured 6 h after adding 0.4 mCi/well. Cells were harvested, and the radioactivity was counted in a scintillation counter (Rack Beta; LKB Wallace). Results were expressed as a ratio of the mean count per minute (cpm) of antigen-stimulated cultures/mean of cpm of unstimulated cultures.

### Co-stimulatory molecule analysis

PBMCs were plated in 24-well plates (5 × 10^5^ cells/ml) then stimulated or not with lipopolysaccharide (LPS) (100 ng/ml) in the presence or absence of the various concentrations of PpSP32 (0.5 μg/ml, 2 μg/ml, 5 μg/ml) for 48 h. After incubation, the cells were washed then labeled with a mixture of specific antibodies each coupled to different fluorochromes: anti-CD14 (FITC), anti-CD86 (APC), and anti-HLA-DR (PE) for 20 min at 4 °C. The cells were then washed and resuspended in a fixing buffer (Cell Fix, Becton Dickinson) until read by the flow cytometer (FACS Canto II, Becton Dickinson).

### Cytokine detection assays

The PBMCs were plated in 24-well plates (5 × 10^5^ cells/ml) then stimulated or not by LPS at 100 ng/ml or PMA/ionomycin at 50 ng/ml and 1 μg/ml, respectively (Sigma). For each condition, the recombinant form of PpSP32 was added with increasing concentrations (0.5 μg/ml, 2 μg/ml, and 5 μg/ml). The plates were placed in an incubator at 37 °C and in the presence of CO_2_ for 48 h. The culture supernatants were then collected and stored at −20 °C until use.

THP-1-derived monocytes were treated or not with different concentrations of PpSP32 (0.5 μg/ml, 2 μg/ml, or 5 μg/ml) for 48 h. Cells were then stimulated with LPS (100 ng/ml) for 18 h. The supernatants were then collected and stored at −20 °C until use.

Enzyme-linked immunosorbent assay (ELISA) was performed on supernatants of PBMCs or THP-1-derived monocytes using human interleukin (IL)-6, interferon-gamma (IFN-γ), or tumor necrosis factor alpha (TNF-α) ELISA sets (BD Biosciences) and IL-1β assay (Human IL-1β Duoset ELISA, R and D systems) according to manufacturer’s instructions. For each cytokine determination, the results were interpolated from a standard curve using recombinant cytokines and expressed in pg/ml.

For some experiments, cytokine multiplex analysis was performed using the human inflammation 11 Plex assay kit [TNF-α, interferon-γ-induced protein-10 (IP-10), IL-1β, IL-27, IFN-γ, IL-8, IL-12p70, monocyte chemoattractant protein-1 (MCP-1), IL-1α, IL-6, IL-10] (Aimplex, Biosciences) according to the manufacturer’s instructions. Briefly, antibody-conjugated capture beads were first incubated with supernatants or standard controls for 60 min, then with biotinylated detection antibodies for 30 min, and finally with streptavidin-PE for 20 min. Fluorescence signals of the beads were acquired by a flow cytometer (FACS Canto II, Becton Dickinson).

### Cell viability assay

The effect of PpSP32 on the viability of THP1-derived macrophages cells was assessed using the 1,3-(4,5-dimethyl-2-thiazolyl)-2,5-diphenyl-2H-tetrazolium bromide) (MTT) method. Differentiated THP-1 cells were seeded at a density of 5 × 10^3^ cells per well in 96-well tissue culture plates and allowed to grow overnight at 37 °C under 5% CO_2_. Cells were cultured for 24 h or 72 h in the presence or not of different concentrations of PpSP32 (0.5 μg/ml, 2 μg/ml, and 5 μg/ml). MTT (0.5 mg/ml) solution was then added, and cells were incubated for a further 3 h. Dimethyl sulfoxide (Sigma) was then added to solubilize formazan crystals, and the optical density was measured at 560 nm to quantify the percentage of living cells. All experiments were performed at least twice in triplicate.

### Western blotting

THP-1-derived macrophages were treated or not with different concentrations of PpSP32 (0.5 μg/ml, 2 μg/ml, and 5 μg/ml) for 48 h at 37 °C, 5% CO_2,_ then stimulated with 100 ng/ml of LPS for 3 h at 37 °C, 5% CO_2._ Total cell lysates were extracted at room temperature with 100 µl of Laemmli buffer (1x) per 5 × 10^5^ cells. Protein concentration was determined by using the Bicinchoninic Acid Protein Assay Kit, (BCA, Sigma) with bovine serum albumin (BSA) as standard. Whole-cell lysates (30 µg/lane) were then separated by sodium dodecyl sulfate polyacrylamide gel electrophoresis (SDS-PAGE) and transferred to a polyvinyl difluoride membrane (PVDF, Amersham). After washing, the membrane was incubated with anti-phospho I kappa B alpha (anti-pIκB-α) antibody (Cell Signaling Technology) at 1:2000 overnight or anti-β actin at 1:1000 (Cell Signaling Technology) for 2 h at room temperature. After washing and incubation with horseradish peroxidase (HRP)-conjugated secondary antibodies (anti-rabbit IgG HRP at 1:2000), immunoblots were determined by enhanced chemiluminescence.

### Carrageenan induced paw edema

The anti-inflammatory activity on carrageenan-induced paw edema was determined according to the method described by Winter et al. [24]. Young adult male rats of 125 to 165 g body weight were maintained in air-conditioned quarters with water and food. Naïve rats were randomly allocated to four groups: the control group received 2.5 ml/kg of physiological solution 0.9% NaCl used to resuspend the different drugs; the standard group received 1 mg/kg of dexamethasone; the positive control group received 15 mg/kg of carrageenan in 100 µl of 0.9% NaCl; and the test group received 15 mg/kg of carrageenan and 5 µg de PpSP32. The drugs were administered into the left hind paw. Edema was followed by measuring changes in paw volumes using a sliding caliper at various times (0, 1, 2, 3, and 4 h). The increase in paw volume was considered as an index of inflammation intensity.

### Statistical analysis

Statistical analyses were performed using one-way analysis of variance (ANOVA) with multiple comparisons when comparing paired groups or the Mann–Whitney test when comparing independent groups. Statistical significance was assigned to a value of *P* < 0.05. All statistical analyses and graphs were performed using GraphPad Prism v5.0 or v8.0 software.

## Results

### Effects of PpSP32 on lymphocyte effector function

**Fig. 1:**
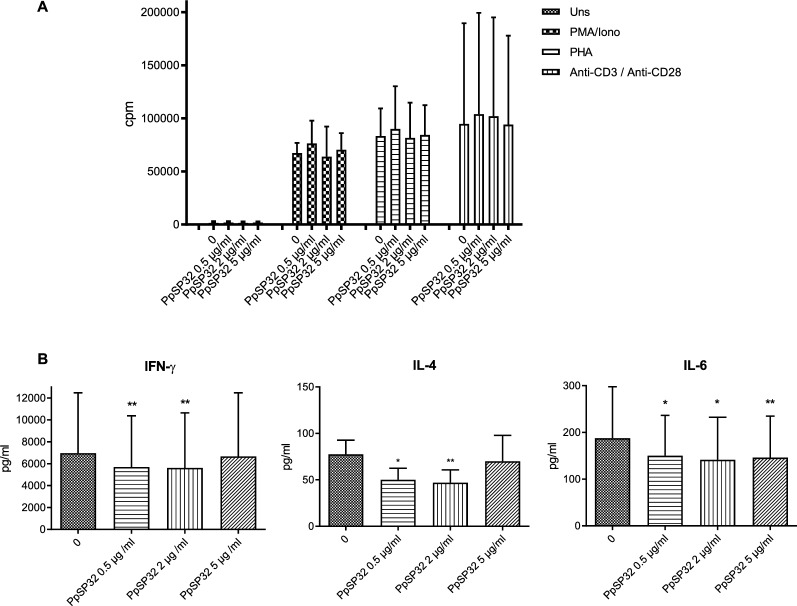
Effects of PpSP32 on lymphocyte effector functions. **A** PBMCs were stimulated or not with PMA/ionomycin, PHA, or anti-CD3/CD28 antibodies in the presence or absence of increasing doses of PpSP32 for 72 h. Proliferative responses were assessed by (3H) thymidine uptake. Results are expressed as count per minute (cpm). Data are means of three independent experiments. **B** PBMCs were stimulated with PMA (50 ng/ml) and ionomycin (1 µg/ml) in the presence or absence of different concentrations of PpSP32 for 48 h. Supernatants were collected and tested for cytokine production by ELISA. Data are presented as means ± SD. The *P*-value was determined by the ANOVA test with multiple comparisons. *Indicates *P* < 0.05, **P < 0.001, ***P < 0.0001. PHA, phytohemagglutinin; PMA, phorbol myristate acetate; Iono, ionomycin; IFN-γ, interferon gamma; Uns, unstimulated; IL, interleukin; pg, picogram; ml, milliliter; µg, microgram; PpSP32, *Phlebotomus papatasi* salivary protein 32

The effect of PpSP32 on lymphocyte effector functions was first assessed by studying their proliferative response to different stimuli (PMA/ionomycin, PHA, and CD3/CD28 antibodies). As shown in Fig. 1A, PpSP32 does not exert any effect on lymphocyte proliferation regardless of the dose of PpSP32 and the type of stimulus used.

When assessing the cytokine production upon activation with PMA/ionomycin, we noted a significant down-modulation of IFN-γ at concentrations of 0.5 µg/ml and 2 µg/ml, IL-4 at concentrations of 0.5 µg/ml and 2 µg/ml, and IL-6 levels at all concentrations used (0.5, 2, and 5 µg/ml) (Fig. 1B).

### Effects of PpSP32 on monocyte effector function

**Fig. 2:**
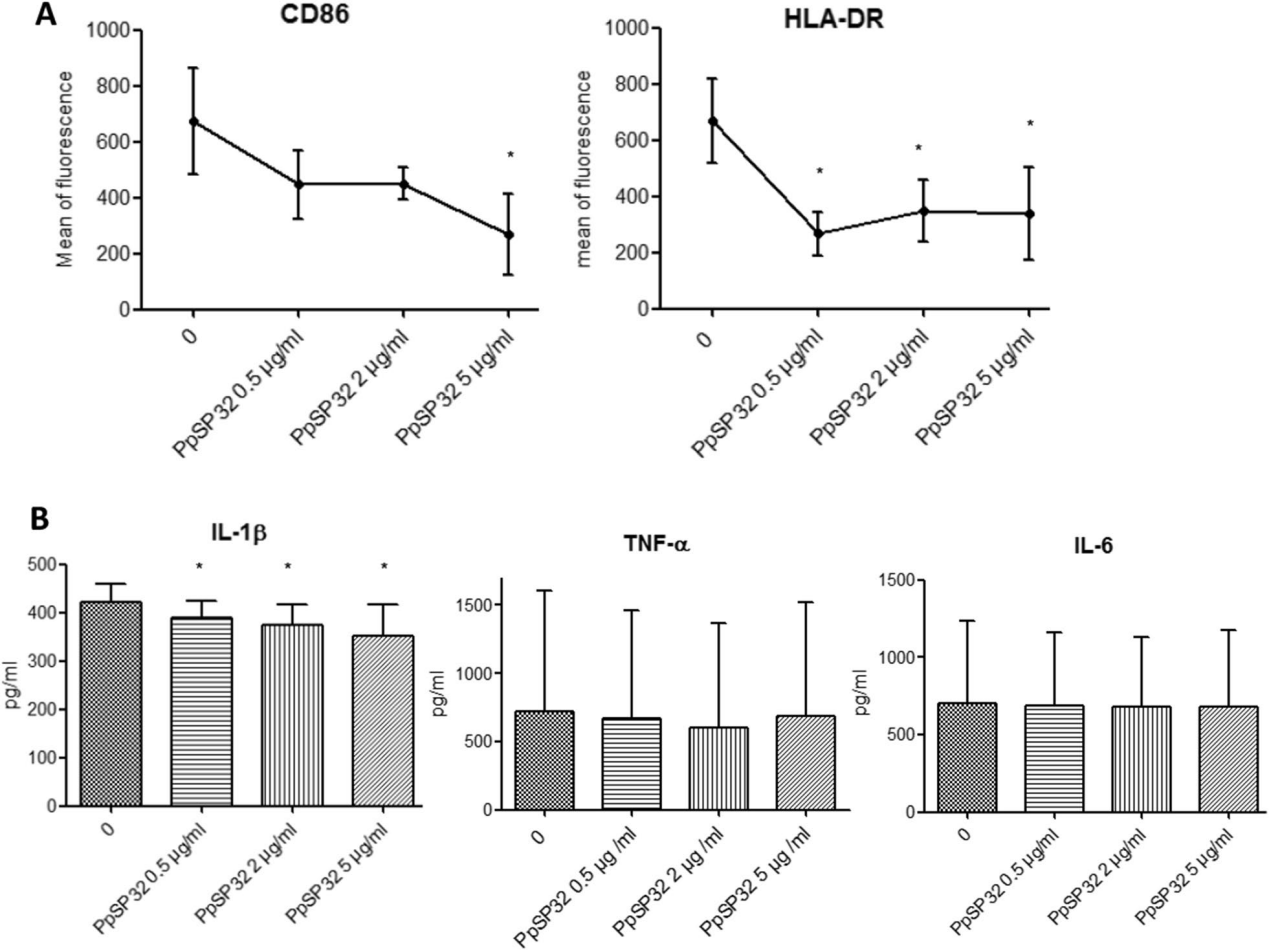
Effects of PpSP32 on monocyte effector function. Peripheral blood mononuclear cells (PBMCs) were stimulated by LPS (100 ng/ml) for 48 h in the presence or not of increasing doses of PpSP32. **A** The mean fluorescence of CD86 and HLA-DR staining in CD14-positive cells is shown. *Indicates *P* < 0.05 when compared to the condition without PpSP32. **B** Cytokine production was tested in the supernatants by ELISA. Data are presented as means ± SD. The *P*-value was determined by the ANOVA test with multiple comparisons. *Indicates *P* < 0.05, **P < 0.001, ***P < 0.0001 when compared to the condition without PpSP32. HLA-DR, human leukocyte antigen receptor; CD86, cluster of differentiation 86; IL, interleukin; TNF-α, tumor necrosis factor alpha; PpSP32, *Phlebotomus papatasi* salivary protein 32; pg, picogram; ml, milliliter; µg, microgram

To test the effect of PpSP32 on monocyte activation, we first assessed the expression of CD86 and HLA-DR surface molecules. PBMCs were thus stimulated with LPS in the presence of increasing doses of PpSP32 (0.5 µg/ml, 2 µg/ml, or 5 µg/ml), and surface molecule expression was analyzed by flow cytometry on CD14+ cells. As shown in Fig. 2A, PpSP32 down-modulated the expression of both surface markers in LPS-stimulated monocytes. Notably, such an effect was dose-dependent for CD86 expression.

In the next step, we analyzed the effect of PpSP32 on the production of pro-inflammatory cytokine upon LPS activation. LPS is a potent stimulus of monocytes and thus represents a more stringent test of the capacity of PpSP32 to modulate monocyte activation. As shown in Fig. 2B, a significant and dose-dependent inhibition of LPS-induced secretion of IL-1β by PpSP32 was observed with all concentrations used (0.5, 2, and 5 µg/ml). The production of TNF-α was slightly but not significantly decreased after the exposure to PpSP32. PpSP32 had no effect on the secretion of IL-6.

### Effects of PpSP32 on THP-1-derived macrophages

The modulatory effects of PpSP32 on monocyte effector function led us to test its effects on THP-1-derived macrophages. The use of cell lines would also overcome the heterogeneity of responses noticed between the different donors.

**Fig. 3:**
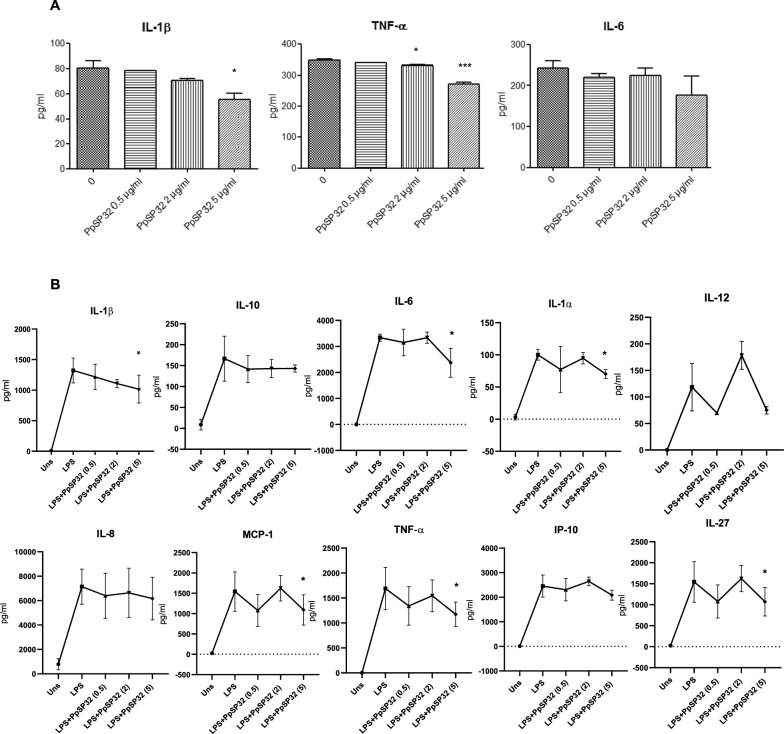
Effects of PpSP32 on cytokine production by THP1-derived macrophages: THP-1-derived macrophages were pretreated with different concentrations of PpSP32, 0.5 µg/ml (0.5), 2 µg/ml (2), and 5 µg/ml (5), for 48 h then stimulated with LPS (100 ng/ml) for 18 h. Supernatants were collected and tested for cytokine production by ELISA (**A**) or by multiplex flow cytometry analysis (**B**). Data are presented as means ± SD. The *P*-value was determined by the ANOVA test with multiple comparisons. *Indicates *P* < 0.05, **P < 0.001, ***P < 0.0001 when compared to the condition without PpSP32. IL, interleukin; TNF-α, tumor necrosis factor alpha; IFN-γ, interferon gamma; MCP-1, monocyte chemoattractant protein 1; IP-10, interferon-γ-induced protein-10; Uns, unstimulated; pg, picogram; ml, milliliter; µg, microgram; PpSP32, *Phlebotomus papatasi* salivary protein 32

We first established the optimal timing for production of IL-1β, IL-6, and TNF-α after LPS stimulation was 18 h in such cell line. The cells were either pretreated or not with different concentrations of PpSp32 for 48 h then stimulated with the LPS for additional 18 h. PpSP32 exhibited an inhibitory effect of all tested cytokines (Fig. 3A). A significant and dose-dependent inhibition by PpSP32 was observed for IL-1β and TNF-α while an inhibition was noticed for IL-6 for the high dose of PpSP32. The inhibitory effect of PpSP32 on pro-inflammatory cytokine production was confirmed by a multiplex assay (Fig. 3B). This effect was noticed also for MCP-1, IP-10, IL-1α, IL-12, IL-8, IL-27, and IL-10, yet it was significant only for MCP-1, IL-27, and IL-1α in addition to IL-1-β, TNF-α, and IL-6. Altogether, these data rather suggest a global inhibitory effect on macrophage cytokine production (Fig. 3B).

**Fig. 4:**
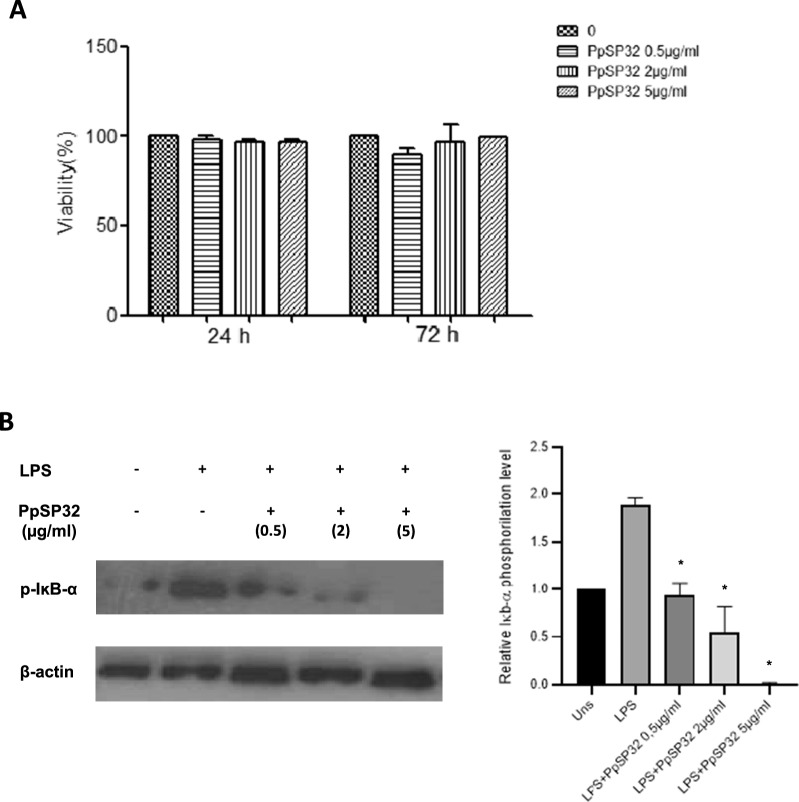
Effects of PpSP32 on human THP-1-derived macrophages. **A** THP-1-derived macrophage viability assessed using the MTT assay was evaluated by incubating cells in the presence or absence of different concentrations of PpSP32 for 24 h and 72 h. Results of three independent experiments are expressed as mean of percentages ± SD of viability according to the control condition. **B** THP-1-derived macrophages were pretreated with PpSP32 (0.5, 2, or 5 μg/ml) for 48 h, then incubated with 100 ng LPS for 3 h. Whole-cell lysates (30 µg/lane) were then separated, transferred, and incubated with anti-p-IκB-α antibody or anti-β actin. Immunoblots were determined by enhanced chemiluminescence after adding the HRP-conjugated secondary antibodies. Quantification of phospho-IκB level was performed by Image J (version 1.8.0). Data are presented as means ± SD. The *P*-value was determined by the ANOVA test with multiple comparisons. *Indicates *P* < 0.05. LPS, lipopolysaccharide; p-IκB-α, phospho I kappa B alpha; Uns, unstimulated; µg, microgram; ml, milliliter; h, hour; PpSP32, *Phlebotomus papatasi* salivary protein 32

To test whether the inhibitory effect of PpSP32 on the pro-inflammatory cytokine production was not related to a reducing cell viability of THP-1-derived macrophages, we used an MTT assay. Our data did not show any significant inhibitory effect on cell growth with different concentrations of PpSP32 after either 24 h or 72 h incubation of THP-1-derived cells (Fig. 4A).

To further confirm the anti-inflammatory effect of PpSP32 on THP-1-derived macrophages, we tested its effect on nuclear factor kappa B (NF-kB) signaling, the main pathway that regulates the expression of many inflammatory cytokines. THP-1 cells were pretreated by increasing doses of PpSP32 then stimulated or not by LPS for 3 h. As shown in Fig. 4B, C, PpSP32 treatment led to a dose-dependent reduction of the IκB phosphorylation.

### Effects of PpSP32 on carrageenan-induced paw edema in rats

**Fig. 5:**
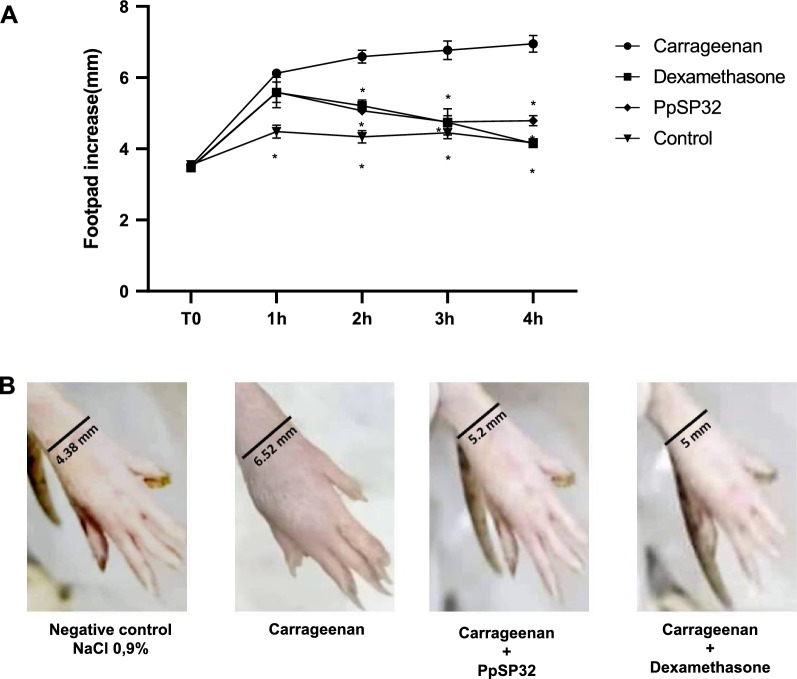
Effects of PpSP32 on carrageenan-induced paw edema in rats. Naïve rats were randomly allocated to four groups of three mice each: the control group received 2.5 ml/kg of physiological solution 0.9% NaCl used to re suspend the different drugs; the standard group received 1 mg/kg of dexamethasone; the positive control group received 15 mg/kg of carrageenan in 100 µl of 0.9% NaCl; and the test group received 15 mg/kg of carrageenan and 5 µg de PpSP32. Edema was followed by measuring changes in paw volumes using a sliding caliper at various times (0, 1, 2, 3, and 4 h). **A** Data are presented as means ± SD. The *P*-value was determined by the Mann–Whitney test. *Indicates *P* < 0.05, **P < 0.001, ***P < 0.0001 when compared to the test group. **B** Pictures from a representative experiment at 3 h of injection are shown. PpSP32, *Phlebotomus papatasi* salivary protein 32; h, hour; mm, millimeter

The carrageenan-induced paw edema model in rats, one of the well-established acute inflammatory models in vivo, was used to test the anti-inflammatory activity of PpSP32. The injection of carrageenan into the rat’s hind paw induced an increase in paw edema, which indicates the development of an inflammatory response. As shown in Fig. 5, the edema was present as early as 1 h after carrageenan injection, progressed rapidly, and persisted for at least 4 h after treatment. Dexamethasone, used as positive control, inhibited the inflammatory response due to carrageenan in rats. Treatment of rats with PpSP32 significantly reduced paw swelling from the second hour. This reduction was about 23% after the second hour and reached a maximum of 30% at the third hour.

## Discussion

This is the first evidence of immunomodulatory functions of PpSP32, the immunodominant salivary protein of *P. papatasi*. It is well known that sand fly saliva contains several molecules which impair the capacity of the hemostatic system by preventing vasoconstriction [9] and inhibiting both platelet aggregation and blood coagulation cascade [25, 26]. Some of these salivary proteins may also interfere with the host’s immune response by inhibiting the complement system or modulating the T cell response or antigen-presenting cell functions [2, 7, 10, 27]. Despite the growing knowledge on the biology of saliva [21, 28, 29], the function of several components, including PpSP32, the immunodominant salivary protein of *P. papatasi* [22], remains unknown to date. PpSP32 harbors structural homology with other proteins such as a flagelliform silk-protein of *N. clavipes* as well as with collagen adhesion proteins [21]. PpSP32 was also predicted to be a mucin-based protein according to the pattern of its O- and N-glycosylation. Interestingly, it was demonstrated that PpSP32 binds to specific proteins from the skin, the desmogleins, leading to the loss of tolerance and the production of related auto-antibodies which causes pemphigus [23]. Herein, we aimed to screen one of the putative effects of this protein, namely the immunomodulatory one.

To evaluate the potential effect of PpSP32 on the cellular immune response, we first assessed its effects on lymphocyte effector functions through testing of the proliferative response as well as the cytokine production of such cells. Although PpSP32 has no effect on the proliferation of T lymphocytes, it exerted an inhibitory effect on Th1 response. Modulation of IFN-γ production following exposure to sand fly saliva has been already described in different species, including *P. papatasi* [15]. Since IFN-γ is a key factor that promotes leishmanicidal mechanisms of macrophages, its inhibition by the sand fly saliva could sustain the multiplication of amastigotes and promote *Leishmania* infection. Our data, however, showed a parallel modulatory effect on Th2 cytokine production. Such results are consistent with those of Rohousova et al., which demonstrated that the salivary gland lysate of three different sand flies, *P. papatasi*, *P. sergenti*, and *Lu. longipalpis*, inhibited the secretion of IL-4 by murine splenocytes [30]. However, another study from Mbow et al. [31] reported a direct enhancing effect of *P. papatasi* saliva on IL-4 expression in the absence of *Leishmania* infection. The latter results were obtained in vivo*,* perhaps explaining the difference with in vitro experiments.

In a second step, the immunomodulatory effect on antigen-presenting cell functions was evaluated through the assessment of the expression of MHC class II and B7 (CD80 and CD86) molecules. In fact, modulation of these surface molecules could alter the response of T lymphocytes and promote tolerance towards *Leishmania* antigens. A significant down-modulation of both molecules was demonstrated, even in the presence of low doses of PpSP32, a result consistent with this previously reported by Costa et al. using the salivary gland homogenate of *Lu. longipalpis* [13]. In the latter report, the effect was mainly ascribed to maxadilan [12]. Contrastingly, in some other species such as *Lu. intermedia*, pretreatment with the salivary gland homogenate was able to significantly increase the expression of CD80, CD86, and HLA-DR on human monocytes [11]. Such discrepancy may be explained by the difference in the composition of such distinct sand fly species, particularly by the difference in the amounts of maxadilan [32].

In a next step, we investigated whether the immune modulation exerted by PpSP32 would affect the secretion of pro-inflammatory cytokines by human monocytes. Our data showed that PpSP32 inhibited IL-1β production by LPS-stimulated PBMCs, but the considerable heterogeneity in responses found in the different donors for TNF-α and IL-6 production hampered the ability to draw strong conclusions. To overcome such issues, we used derived macrophages from the THP-1 cell line [33–35] and confirmed the inhibition of IL-1β, TNF-α, and IL-6 in the latter cells. IL-1β and TNF-α play a crucial role in the clearance of *Leishmania* infection [36, 37]. Downregulation of TNF-α has also been reported for other components of saliva, such adenosine, a pharmacologically active component of *P. papatasi* saliva [38, 39], maxadilan, a salivary component of the sand fly *Lu. longipalpis* [15], or RsP03 of *P. perniciosus* [40]. Interestingly, down-modulation of IL-6 production exerted by PpSP32 contrasts with several reports in the literature regarding other salivary sand fly components. Accordingly, either maxadilan, *P. papatasi*, *P. alexandri*, or *P. duboscqi* salivary extracts have been reported to enhance the production of IL-6 by monocytes or PBMCs [15, 39, 40]. To the best of our knowledge, this is the first report revealing a downregulation of IL-6 by a salivary protein of *P. papatasi.* This down-modulation has been shown in our work not only in LPS-stimulated monocytes/macrophages but also in PMA-ionomycin-stimulated lymphocytes.

In addition to TNF-α and IL-1β, a number of other inflammatory cytokines are involved in macrophage functions [41]. Hence, a multiplex technique was used to measure several inflammatory cytokines after stimulation of THP-1-derived macrophages in the presence of different concentrations of PpSP32. Our results showed that PpSP32 decreases the secretion of IL-6, TNF-α, and IL-1β, and also IL-1α, IL-12, IL-27, IL-8, IP10, MCP1, and IL-10, yet a significant effect was reported only with for MCP-1, IL-27, and IL-1α, in addition to TNF-α, IL-1β, and IL-6. It is well known that IL-12, together with IFN-γ, participates in the polarization of the immune response by inducing the commitment of naive T cells into Th1 cells while inhibiting the development of Th2 lymphocytes [42]. Our data are consistent with those of Mbow et al. [31] who reported that salivary gland homogenate of *P. papatasi* inhibits the expression of IL-12 in mice. Since Link et al. [43] demonstrated that adenosine from *P. papatasi* saliva inhibited IL-12 secretion by human monocytes, we can suggest that the inhibition of IL-12 by *P. papatasi* sand fly saliva could be ascribed to PpSP32 along with adenosine.

Primarily expressed by macrophages and dendritic cells during the early phase of *Leishmania* infection, IL-27 contributes to protection against *L. major* infection. This protection is exerted primarily through inhibition of IL-4-mediated Th2 cell responses and induction of IFN-γ production from CD4+ T and NK cells, thereby enhancing Th1 cell responses [44]. Furthermore, IL-27 enhances the differentiation of monocytes into macrophages and activates macrophages [45]. To our knowledge, this is the first report which studies the effect of a salivary protein on IL-27 secretion by monocyte-derived macrophages and which demonstrated a decrease in the secretion of this cytokine after PpSP32 stimulation.

IL-8, IP-10, and MCP-1 are potent chemotactic agents responsible for migration of several immune cells [46, 47]. Our results showed that PpSP32 decreased the secretion of all of them, particularly MCP-1. A previous report showed that pre-exposure of murine cells to *Lu. intermedia* salivary sonicates resulted in decreased expression of IP-10 during subsequent secondary exposures [48]. Another report showed that the saliva of *Lu. longipalpis* seems to increase levels of IL-8 secreted by LPS-stimulated human monocytes [13]. One work studied the effect of sand fly salivary components on MCP-1 expression, but this report tested this expression by neutrophils and not macrophages. It revealed that *Lu. longipalpis* saliva in the presence of *L. chagasi* induces MCP-1 expression by neutrophils, which contrasts with our data showing an inhibitory effect of PpSP32 on these cytokines in monocyte/macrophages [49].

Contrasting with a global anti-inflammatory effect of PpSP32, our data showed that this molecule exerted an inhibitory effect also on IL-10, an anti-inflammatory cytokine. Divergent results have been reported regarding the effect of salivary components of sand fly saliva on IL-10 production. While *Lu. longipalpis* saliva decreases IL-10 secretion by human monocytes stimulated with LPS [13], adenosine and its precursor 5′-AMP, isolated from *P. papatasi* salivary glands, have been reported to enhance IL-10 [43]. Maxadilan also stimulates the secretion of IL-10 by macrophages [50]. Regarding our data about the effects of PpSP32 on the cytokine production by monocytes/macrophages, the parallel inhibitory effect on either pro-inflammatory or anti-inflammatory mediators is intriguing. This could rather point to an overall inhibitory effect on monocyte/macrophage functions, since IL-10 is also produced by these cells after activation. These findings also argue against the fact that the inhibitory effect exerted by PpSP32 on monocyte/macrophage function is mediated by the overproduction of IL-10.

Overall, our study provides new insights into the effects of PpSP32 on cytokine production by human PBMCs as well as by monocytes and human THP-1-derived macrophages. Herein, we showed for the first time that PpSP32 possesses rather anti-inflammatory properties. *Leishmania* parasites appear to exploit these immunomodulatory properties of PpSP32 to enhance their early survival in humans and exacerbate infection. To better understand the mechanisms underlying our observations and make these findings more convincing, we investigated the mechanism by which PpSP32 modulates cytokine secretion. Hence, the effect of PpSP32 pretreatment on the NFκB signaling pathway was assessed by testing the phosphorylation of IκB in LPS-stimulated THP-1 cells [51–53]. Our findings suggested for the first time that PpSP32 inhibited LPS-induced inflammatory response in THP-1 cells through the inhibition of the NFκB signaling pathway. However, we could not exclude that other cellular pathways may be involved in the biological activity of PpSP32.

Finally, the anti-inflammatory activity of PpSP32 was further assessed in vivo in an experimental inflammatory model, the carrageenan-induced paw edema in rats. The injection of carrageenan produces time- and dose-dependent edema in the paws of rats. Interestingly, when PpSP32 was injected into the paw, edema was reduced in the same manner as dexamethasone, a corticosteroid and anti-inflammatory molecule. The in vivo inhibition of inflammation by salivary proteins has been already reported. Lufaxin, a novel factor Xa inhibitor from the salivary gland of *Lu. longipalpis* has been demonstrated to inhibit inflammation in vivo. In fact, lufaxin abrogates edema formation triggered by injection of factor Xa in the paw of mice [54]. In vivo sub-plantar injection of this factor induces edema in the paws of mice that resembles the effects observed after administration of carrageenan [55].

## Conclusion

This study indicates for the first time that PpSP32 induces a potent immunomodulatory effect on monocytes and THP-1-derived macrophages. This inhibition could be mediated, among others, through the modulation of the NF-kB signaling pathway. The underlying mechanisms are under study. Our study is a first step in understanding the functions of the immunodominant salivary protein of *P. papatasi*, PpSP32. The investigation of other biological functions of PpSP32 such as anti-hemostatic effects is in progress in order to complete the overall picture.

## Data Availability

Supporting data for the conclusions of this article are included within the article. The raw datasets used and/or analyzed during the current study are available from the corresponding author on reasonable request.

## Abbreviations

APC: Allophycocyanin
ATCC: American Type Culture Collection
ELISA: Enzyme-linked immunosorbent assay
FITC: Fluorescein isothiocyanate
HLA-DR: Human leukocyte antigen receptor
HRP: Horseradish peroxidase
IFN-γ: Interferon gamma
IL: Interleukin
IP-10: Interferon-γ-induced protein-10
LPS: Lipopolysaccharide
*Lu*: *Lutzomyia*
MCP-1: Monocyte chemoattractant protein 1
MTT: 1,3-(4,5-Dimethyl-2-thiazolyl)-2,5-diphenyl-2H-tetrazolium bromide)
NF-kB: Nuclear factor kappa B
*P*: *Phlebotomus*
PBMC: Human peripheral blood mononuclear cells
PE: Phycoerythrin
PHA: Phytohemagglutinin
p-IκB-α: Phospho I kappa B-alpha
PMA: Phorbol 12-myristate 13-acetate
PpSP32: *Phlebotomus papatasi* salivary protein 32
PVDF: Polyvinylidene fluoride
RPMI: Roswell Park Memorial Institute medium
SDS–PAGE: Sodium dodecyl sulfate–polyacrylamide gel electrophoresis
TNF-α: Tumor necrosis factor alpha

## References

[1] Ribeiro JM (1995). Blood-feeding arthropods: live syringes or invertebrate pharmacologists?. Infect Agents Dis.

[2] Andrade BB, Teixeira CR, Barral A, Barral-Netto M (2005). Haematophagous arthropod saliva and host defense system: a tale of tear and blood. An Acad Bras Cienc.

[3] Titus RG, Ribeiro JM (1988). Salivary gland lysates from the sand fly *Lutzomyia longipalpis* enhance *Leishmania* infectivity. Science.

[4] Belkaid Y, Kamhawi S, Modi G, Valenzuela J, Noben-Trauth N, Rowton E, Ribeiro J, Sacks DL (1998). Development of a natural model of cutaneous leishmaniasis: powerful effects of vector saliva and saliva preexposure on the long-term outcome of *Leishmania* major infection in the mouse ear dermis. J Exp Med.

[5] Morris RV, Shoemaker CB, David JR, Lanzaro GC, Titus RG (2001). Sandfly maxadilan exacerbates infection with *Leishmania* major and vaccinating against it protects against L. major infection. J Immunol.

[6] de Moura TR, Oliveira F, Novais FO, Miranda JC, Clarêncio J, Follador I, Carvalho EM, Valenzuela JG, Barral-Netto M, Barral A, Brodskyn C (2007). Enhanced *Leishmania braziliensis* infection following pre-exposure to sandfly saliva. PLoS Negl Trop Dis.

[7] Lestinova T, Rohousova I, Sima M, de Oliveira CI, Volf P (2017). Insights into the sand fly saliva: blood-feeding and immune interactions between sand flies, hosts, and *Leishmania*. PLoS Negl Trop Dis.

[8] Lerner EA, Ribeiro JM, Nelson RJ, Lerner MR (1991). Isolation of maxadilan, a potent vasodilatory peptide from the salivary glands of the sand fly *Lutzomyia longipalpis*. J Biol Chem.

[9] Ribeiro JM, Katz O, Pannell LK, Waitumbi J, Warburg A (1999). Salivary glands of the sand fly *Phlebotomus papatasi* contain pharmacologically active amounts of adenosine and 5′-AMP. J Exp Biol.

[10] Feitosa IB, de Aguida WR, Teles CBG (2018). EntomoBrasilis. EntomoBrasilis.

[11] Menezes MJ, Costa DJ, Clarêncio J, Miranda JC, Barral A, Barral-Netto M, Brodskyn C, de Oliveira CI (2008). Immunomodulation of human monocytes following exposure to *Lutzomyia intermedia* saliva. BMC Immunol.

[12] Wheat WH, Pauken KE, Morris RV, Titus RG (2008). *Lutzomyia longipalpis* salivary peptide maxadilan alters murine dendritic cell expression of CD80/86, CCR7, and cytokine secretion and reprograms dendritic cell-mediated cytokine release from cultures containing allogeneic T cells. J Immunol.

[13] Costa DJ, Favali C, Clarêncio J, Afonso L, Conceição V, Miranda JC, Titus RG, Valenzuela J, Barral-Netto M, Barral A, Brodskyn CI (2004). *Lutzomyia longipalpis* salivary gland homogenate impairs cytokine production and costimulatory molecule expression on human monocytes and dendritic cells. Infect Immun.

[14] Cavalcante RR, Pereira MH, Gontijo NF (2003). Anti-complement activity in the saliva of phlebotomine sand flies and other haematophagous insects. Parasitology.

[15] Rogers KA, Titus RG (2003). Immunomodulatory effects of Maxadilan and *Phlebotomus papatasi* sand fly salivary gland lysates on human primary in vitro immune responses. Parasite Immunol.

[16] Arcà B, Ribeiro JM (2018). Saliva of hematophagous insects: a multifaceted toolkit. Curr Opin Insect Sci.

[17] Wikel S, Wikel SK, Aksoy S, Dimopoulos G (2017). Chapter 3—Arthropod modulation of wound healing. Arthropod vector: controller of disease transmission.

[18] Postigo JAR (2010). Leishmaniasis in the World Health Organization Eastern Mediterranean Region. Int J Antimicrob Agents.

[19] Abdeladhim M, Kamhawi S, Valenzuela JG (2014). What’s behind a sand fly bite? The profound effect of sand fly saliva on host hemostasis, inflammation and immunity. Infect Genet Evol.

[20] Valenzuela JG, Belkaid Y, Garfield MK, Mendez S, Kamhawi S, Rowton ED, Sacks DL, Ribeiro J (2001). Toward a defined anti-*Leishmania* vaccine targeting vector antigens: characterization of a protective salivary protein. J Exp Med.

[21] Hostomská J, Volfová V, Mu J, Garfield M, Rohousová I, Volf P, Valenzuela JG, Jochim RC (2009). Analysis of salivary transcripts and antigens of the sand fly *Phlebotomus arabicus*. BMC Genomics.

[22] Marzouki S, Abdeladhim M, Abdessalem CB, Oliveira F, Ferjani B, Gilmore D, Louzir H, Valenzuela JG, Ahmed MB (2012). Salivary antigen SP32 is the immunodominant target of the antibody response to *Phlebotomus papatasi* bites in humans. PLoS Negl Trop Dis.

[23] Marzouki S, Zaraa I, Abdeladhim M, Benabdesselem C, Oliveira F, Kamhawi S, Mokni M, Louzir H, Valenzuela JG, Ahmed MB (2020). Implicating bites from a leishmaniasis sand fly vector in the loss of tolerance in pemphigus. JCI Insight.

[24] Winter CA, Risley EA, Nuss GW (1962). Carrageenin-induced edema in hind paw of the rat as an assay for antiiflammatory drugs. Proc Soc Exp Biol Med.

[25] Koh CY, Kini RM (2009). Molecular diversity of anticoagulants from haematophagous animals. Thromb Haemost.

[26] Francischetti IMB (2010). Platelet aggregation inhibitors from hematophagous animals. Toxicon.

[27] Oliveira F, Traoré B, Gomes R, Faye O, Gilmore DC, Keita S, Traoré P, Teixeira C, Coulibaly CA, Samake S, Meneses C (2013). Delayed-type hypersensitivity to sand fly saliva in humans from a leishmaniasis-endemic area of Mali is Th1-mediated and persists to midlife. J Investig Dermatol.

[28] Wahba M, Riera C (2006). Salivary gland composition of some old world vector sand fly. J Egypt Soc Parasitol.

[29] Coutinho-Abreu IV, Guimaraes-Costa AB, Valenzuela JG (2015). Impact of insect salivary proteins in blood feeding, host immunity, disease, and in the development of biomarkers for vector exposure. Curr Opin Insect Sci.

[30] Rohousová I, Volf P, Lipoldová M (2005). Modulation of murine cellular immune response and cytokine production by salivary gland lysate of three sand fly species. Parasite Immunol.

[31] Mbow ML, Bleyenberg JA, Hall LR, Titus RG (1998). *Phlebotomus papatasi* sand fly salivary gland lysate down-regulates a Th1, but up-regulates a Th2, response in mice infected with *Leishmania* major. J Immunol.

[32] de Moura TR, Oliveira F, Carneiro MW, Miranda JC, Clarêncio J, Barral-Netto M, Brodskyn C, Barral A, Ribeiro JM, Valenzuela JG, de Oliveira CI (2013). Functional transcriptomics of wild-caught *Lutzomyia intermedia* salivary glands: identification of a protective salivary protein against *Leishmania braziliensis* infection. PLoS Negl Trop Dis.

[33] Schwende H, Fitzke E, Ambs P, Dieter P (1996). Differences in the state of differentiation of THP-1 cells induced by phorbol ester and 1,25-dihydroxyvitamin D3. J Leukoc Biol.

[34] Daigneault M, Preston JA, Marriott HM, Whyte MKB, Dockrell DH (2010). The identification of markers of macrophage differentiation in PMA-stimulated THP-1 cells and monocyte-derived macrophages. PLoS ONE.

[35] Tsuchiya S, Yamabe M, Yamaguchi Y, Kobayashi Y, Konno T, Tada K (1980). Establishment and characterization of a human acute monocytic leukemia cell line (THP-1). Int J Cancer.

[36] Rohousová I, Volf P (2006). Sand fly saliva: effects on host immune response and *Leishmania* transmission. Folia Parasitol.

[37] Kaneko N, Kurata M, Yamamoto T, Morikawa S, Masumoto J (2019). The role of interleukin-1 in general pathology. Inflamm Regen.

[38] Haskó G, Szabó C, Németh ZH, Kvetan V, Pastores SM, Vizi ES (1996). Adenosine receptor agonists differentially regulate IL-10, TNF-alpha, and nitric oxide production in RAW 264.7 macrophages and in endotoxemic mice. J Immunol.

[39] Haskó G, Kuhel DG, Chen JF, Schwarzschild MA, Deitch EA, Mabley JG, Marton A, Szabó C (2000). Adenosine inhibits IL-12 and TNF-[alpha] production via adenosine A2a receptor-dependent and independent mechanisms. FASEB J.

[40] Sumova P, Polanska N, Lestinova T, Spitzova T, Kalouskova B, Vanek O, Volf P, Rohousova I (2020). *Phlebotomus perniciosus* recombinant salivary proteins polarize murine macrophages toward the anti-inflammatory phenotype. Front Cell Infect Microbiol.

[41] Arango Duque G, Descoteaux A (2014). Macrophage cytokines: involvement in immunity and infectious diseases. Front Immunol.

[42] Gee K, Guzzo C, Che Mat NF, Ma W, Kumar A (2009). The IL-12 family of cytokines in infection, inflammation and autoimmune disorders. Inflamm Allergy Drug Targets.

[43] Link AA, Kino T, Worth JA, McGuire JL, Crane ML, Chrousos GP, Wilder RL, Elenkov IJ (2000). Ligand-activation of the adenosine A2a receptors inhibits IL-12 production by human monocytes. J Immunol.

[44] Jafarzadeh A, Nemati M, Chauhan P, Patidar A, Sarkar A, Sharifi I, Saha B (2020). Interleukin-27 functional duality balances *Leishmania* infectivity and pathogenesis. Front Immunol.

[45] Abdalla AE, Li Q, Xie L, Xie J (2015). Biology of IL-27 and its role in the host immunity against *Mycobacterium tuberculosis*. Int J Biol Sci.

[46] Yoshimura T, Yuhki N, Moore SK, Appella E, Lerman MI, Leonard EJ (1989). Human monocyte chemoattractant protein-1 (MCP-1). Full-length cDNA cloning, expression in mitogen-stimulated blood mononuclear leukocytes, and sequence similarity to mouse competence gene JE. FEBS Lett.

[47] Mukaida N, Harada A, Matsushima K (1998). Interleukin-8 (IL-8) and monocyte chemotactic and activating factor (MCAF/MCP-1), chemokines essentially involved in inflammatory and immune reactions. Cytokine Growth Factor Rev.

[48] de Moura TR, Oliveira F, Rodrigues GC, Carneiro MW, Fukutani KF, Novais FO, Miranda JC, Barral-Netto M, Brodskyn C, Barral A, de Oliveira CI (2010). Immunity to *Lutzomyia intermedia* saliva modulates the inflammatory environment induced by *Leishmania braziliensis*. PLoS Negl Trop Dis.

[49] Prates DB, Araújo-Santos T, Luz NF, Andrade BB, França-Costa J, Afonso L, Clarêncio J, Miranda JC, Bozza PT, DosReis GA, Brodskyn C (2011). *Lutzomyia longipalpis* saliva drives apoptosis and enhances parasite burden in neutrophils. J Leukoc Biol.

[50] Bozza M, Soares MB, Bozza PT, Satoskar AR, Diacovo TG, Brombacher F, Titus RG, Shoemaker CB, David JR (1998). The PACAP-type I receptor agonist maxadilan from sand fly saliva protects mice against lethal endotoxemia by a mechanism partially dependent on IL-10. Eur J Immunol.

[51] Bondeson J, Foxwell B, Brennan F, Feldmann M (1999). Defining therapeutic targets by using adenovirus: blocking NF-kappaB inhibits both inflammatory and destructive mechanisms in rheumatoid synovium but spares anti-inflammatory mediators. Proc Natl Acad Sci U S A.

[52] Trede NS, Tsytsykova AV, Chatila T, Goldfeld AE, Geha RS (1995). Transcriptional activation of the human TNF-alpha promoter by superantigen in human monocytic cells: role of NF-kappa B. J Immunol.

[53] Andreakos E, Udalova I, Sacre S, Foxwell BM, Beyaert R (2003). The role of NF-κB in inflammatory diseases. Nuclear factor кB: regulation and role in disease.

[54] Collin N, Assumpção TCF, Mizurini DM, Gilmore DC, Dutra-Oliveira A, Kotsyfakis M, Sá-Nunes A, Teixeira C, Ribeiro JM, Monteiro RQ, Valenzuela JG (2012). Lufaxin, a novel factor Xa inhibitor from the salivary gland of the sand fly *Lutzomyia longipalpis* blocks protease-activated receptor 2 activation and inhibits inflammation and thrombosis in vivo. ArteriosclerThrombVasc Biol.

[55] Cirino G, Cicala C, Bucci M, Sorrentino L, Ambrosini G, DeDominicis G, Altieri DC (1997). Factor Xa as an interface between coagulation and inflammation. Molecular mimicry of factor Xa association with effector cell protease receptor-1 induces acute inflammation in vivo. J Clin Investig.

